#  Circulatory disease mortality among male medical radiation workers in South Korea, 1996–2019

**DOI:** 10.5271/sjweh.4066

**Published:** 2023-02-27

**Authors:** Ye Jin Bang, Young Min Kim, Won Jin Lee

**Affiliations:** 1 Department of Preventive Medicine, Korea University College of Medicine, Seoul, South Korea; 2 Graduate School of Public Health, Korea University, Seoul, South Korea.; 3 Department of Statistics, Kyungpook National University, Daegu, South Korea

**Keywords:** cardiovascular disease, cerebrovascular disease, hospital worker, ionizing radiation, ischemic heart disease, occupational exposure

## Abstract

**Objective:**

The aim of this study was to investigate the relationship between occupational radiation exposure and circulatory disease (CD) mortality among medical radiation workers.

**Methods:**

The study included 53 860 male diagnostic medical radiation workers enrolled in the National Dosimetry Registry (NDR) between 1996 and 2011 in South Korea. NDR data were linked with mortality data obtained from the national registry at the end of 2019. Observed CD mortality rates in this population were compared to those in the general population using the standardized mortality ratio (SMR). The relative risk (RR) for occupational history was estimated by use of internal comparisons, and the excess relative risk (ERR) was used to quantify the radiation dose–response relationship.

**Results:**

A total of 320 deaths due to CD were identified among 53 860 male medical radiation workers. The SMR of CD was significantly lower among male workers than the general population. A linear dose–response model provided an estimated ERR per 100 mGy for CD [0.85, 95% confidence interval (CI) -0.11–1.82], ischemic heart disease (1.18, 95% CI -0.69–3.05), and cerebrovascular disease (0.23, 95% CI -0.48–0.94) with a 10-years lag, showing no statistical evidence of a radiation dose–response relationship. Additional adjustments for non-radiation factors did not affect the findings on occupational radiation risk for CD mortality. Sensitivity analyses excluding workers employed <1 year or who had exposure to a cumulative badge dose of ≥1 mSv showed similar results.

**Conclusions:**

Occupational radiation doses were non-significantly positively associated with CD mortality among male diagnostic medical radiation workers. However, cautious interpretation is needed due to the limitations of short follow-up.

Circulatory disease (CD) is a leading cause of death worldwide, and the burden continues to increase globally (1). An estimated 17.9 million people died from cardiovascular diseases in 2019, representing 32% of global deaths (2). The age-dependent distribution of cardiovascular risk factors implies that with the current trend in favor of aging populations, cardiovascular diseases will have an even greater burden in the future. High-dose ionizing radiation is an important risk factor for CD (3, 4). Previous reviews have reported evidence showing a positive association of radiation exposure and CD mortality (5–7).

However, the evidence for an association between low-dose and low-dose-rate ionizing radiation exposure and CD remains controversial, in particular because of the uncertain influence of major non-radiation risk factors on the reported associations (8). Given the high background rates of CD, the estimates of overall radiation-related mortality may be about twice that of current estimates based on radiation-induced cancers alone (5). While the major focus on radiation epidemiology to date has been on cancer, research on radiation-induced CD would contribute to evaluating scientific and social priority concerning chronic exposure in humans.

Studies on occupational radiation workers are valuable sources of information on the risk factors for CD from chronic low-dose radiation exposure. However, only a few epidemiological studies have focused on cohorts of medical radiation workers exposed to X-ray ionizing radiation (9–12). Additionally, occupationally received doses for individual organs have rarely been estimated for medical personnel. However, medical radiation workers receive information on exposure doses on a regular basis, and they have the advantage of the opportunity to evaluate the radiation dose–response relationship. Therefore, epidemiological studies of radiation workers will increase our understanding of the health effects of protracted exposure to low-level radiation (13).

We constructed a registry-based cohort by combining information on all diagnostic medical radiation workers enrolled in the South Korean National Dose Registry (NDR) between 1996 and 2011 with the national mortality data. A general description of mortality in this cohort has been previously reported (14). We extended this study by linking mortality data with coverage through the end of 2019 to investigate the role of occupational radiation exposure in CD deaths. This report compared CD mortality rates among South Korean diagnostic medical radiation workers to those seen in the general population and examined the evidence for the effects of occupational characteristics and radiation doses on CD deaths in the cohort.

## Methods

### Study population and ascertainment of circulatory disease

The details of the study population and survey methodology for Korean diagnostic medical radiation workers have been published previously (14). Briefly, the study cohort comprised 94 394 diagnostic medical radiation workers enrolled in the NDR between 1 January 1996 and 31 December 2011. Personal identification numbers were sent to Statistics Korea, which then linked these numbers to mortality data to ascertain the cause of death among study participants. The uniqueness of individual personal identification numbers in South Korea makes this linkage specific. Statistics Korea has maintained an almost 100% level of completeness (http://kostat.go.kr). Mortality data were classified according to the underlying cause of death as determined by the International Classification of Diseases and Related Health Problems, 10th revision (ICD-10 code). CD (ICD-10 I00-I99) were divided into two major groups: ischemic heart disease (IHD) (ICD-10 I20-I25) and cerebrovascular disease (CeVD) (ICD-10 I60-I69). Participants with incomplete personal identification numbers (N=18) were excluded.

Female workers were initially followed-up, but they were ultimately excluded from the analysis because small numbers of CD-related death cases were noted among them (N=14). Therefore, the final analysis of this study included 53 860 male workers. The Korea University Institutional Review Board reviewed and approved this study (KUIRB-2019-0092-06).

### Estimation of radiation dose

NDR is a government-operated centralized dosimetry data registry for diagnostic radiation workers that has been in operation since 1996. The Korea Disease Control and Prevention Agency (KDCA) maintains the system for all diagnostic radiation medical workers in South Korea (15). For evaluating personal occupational radiation dose, historical dose reconstruction was performed for workers who began working with radiation prior to 1996 using an annual dose model that described doses as a log-linear function of calendar year and age at the year of exposure (16). Organ-specific doses were estimated as the product of individual badge doses and conversion coefficients provided by the National Council on Radiation Protection and Measurements (NCRP): organ-specific conversion coefficient (gray/sievert) for heart and thyroid doses in males were 0.50 and 0.96, respectively, at exposure scenarios involving the use of X-rays in a medical setting (17). Heart dose was used for the risk estimation of CD and IHD, while thyroid dose was used for CeVD risk estimation (6).

### Imputation of non-radiation risk factors

Literature on radiation epidemiological studies was used to select a few non-radiation risk factors for CD (smoking status, alcohol intake, sleep duration, and shift work) (18–20). The findings of previously self-reported questionnaires among diagnostic medical radiation workers from 2012–2013 (response rate was 26.5%) (21), were used as the basis to imput these non-radiation risk factors for male workers. We conducted micro-level data integration of NDR and survey data, and imputed missing values using the multiple imputations by chained equations (MICE) algorithm (22). In this study, we assumed that the missing values were random. MICE is based on a variable-by-variable basis and imputes missing data multiple times in a dataset through an iterative procedure.

### Statistical analyses

Each person contributed person-years at risk from 1996 or the year they started working, based on the NDR, whichever occurred later. The end of follow-up was considered the earliest of the following: date of death or 31 December 2019. The DATAB module in Epicure software was used to create a person-year table stratified by attained age (<40, 5-year intervals from age 40–84, ≥85 years), calendar year (1996–2000, 2001–2005, 2006–2010, 2011–2015, 2016–2019), job title (physician, non-physician), types of medical facility (general hospital, hospital and clinic, dental hospital and clinic, others), area of medical facility (metropolitan, city, rural), birth year (<1960, 1960–1969, ≥1970), calendar year of work begun (<1996, 1996–2004, ≥2005), years of employment duration (< 1, 1–4, 5–9, ≥10), cumulative badge dose (<1, 1–4, 5–19, 20–49, ≥50 mSv), smoking status (never smoker, former smoker, current smoker <12.5, current smoker ≥12.5 pack-years, unknown), alcohol intake (never drinker, ≤1, 2–3, ≥4 per month, unknown), sleep duration (<7, ≥7hour per day, unknown), and shift work (never, ever, unknown).

The standardized mortality ratio (SMR) and the corresponding 95% confidence intervals (CI) for CD were calculated by the Poisson regression method using the South Korean mortality rates. The expected number of deaths and CD deaths for each cell were computed as the product of the number of person-years and the age-, and calendar-year-specific male South Korean mortality rates (http://kostat.go.kr). The relative risk (RR) and corresponding 95% CI were calculated by Poisson regression using the maximum likelihood method to explore the relationship between occupational characteristics and the mortality of CD. The linear trends of RR with cumulative badge dose categories were examined using simple dose–response models. We also examined the RR adjusted for lifestyle variables obtained from the imputation.

The excess relative risk (ERR) and 95% CI for CD mortality were calculated using Poisson regression to analyze the relationship between cumulative organ doses and CD deaths. The primary model used to evaluate the dose–response relationship assumed a linear dose– response relationship. The linear model was written as RR=1 + βd, where RR is the relative risk, d is the dose, and β is an estimate of the excess relative risk per unit dose (ERR/100 mGy). Parameter estimates and 95% CI were calculated using the maximum likelihood method. Variables were selected based on each model’s deviance and Akaike information criteria. Cumulative absorbed organ doses were treated as a time-dependent variable which was lagged by ten years; this lag period is commonly used in other studies of CD in radiation workers, to account for the latency period between exposure and CD onset. The final models were adjusted for attained age, birth year, and number of years of employment in a person-year table and stratified by the factors previously described. ERR models with linear, quadratic, and quadratic terms did not improve the model fit (supplementary material, www.sjweh.fi/article/4066, table S1).

Sensitivity analyses for ERR were conducted for workers: (i) whose employment began after 1995 (N=44 019) to reduce the uncertainties for dose reconstruction; (ii) who were employed for ≥1 year (N=46 366) to avoid possible heterogeneity of the subjects; and (iii) who had a cumulative badge dose of ≥1 mSv (N=31 641) to focus on workers who had greater exposure. We also examined variations in baseline rates and radiation risks by job title (physician versus non-physician) and conducted additional analyses using alternative lagged cumulative organ doses (ie, no lag, 5-years, and 15-years). For adjustment of lifestyle variables, such as smoking status, alcohol intake, sleep duration, and shift work obtained from imputation, we estimated the point estimates for each RR and ERR from the 20 pseudo-complete datasets in the imputation stage. The integrated point estimate and 95% CI were calculated using the arithmetic mean. All analyses were conducted using the AMFIT module in Epicure (Risk Sciences International, version 2.0; Ottawa, Canada).

## Results

The demographic and occupational characteristics of the study population are shown in table 1. Out of 53 860 male medical radiation workers, comprising 893 496 person-years of follow-up, 1658 total deaths and 320 deaths (19.3% of the total deaths) were registered with CD as the underlying cause of death. Of these, 124 deaths were due to IHD while 98 deaths were due to CeVD. CD deaths were older, began working at younger ages, and had worked for a longer duration than those who are still alive. The mean attained age at the end of follow-up was 63.1 years. The mean cumulative badge doses among the CD deaths and workers who are alive were 27.1 mSv and 10.2 mSv, respectively. The estimated heart and thyroid doses were higher in CD deaths than in workers who are still alive. Workers who died from CD were more likely to be smokers in comparison to workers who are still living.

**Table 1:** Occupational characteristics by cause of death among male diagnostic medical radiation workers in South Korea, 1996–2019. [CD=circulatory disease; CeVD=cerebrovascular disease; IHD=ischemic heart disease].

	Total alive (N=52 202)		Total deaths (N=1658)		CD (N=320)		IHD (N=124)		CeVD (N=98)	
				
	N (%)	Mean (min-max)	N (%)	Mean (min-max)	N (%)	Mean (min-max)	N (%)	Mean (min-max)	N (%)	Mean (min-max)
Job title									
Physician	28 415 (54.4)		958 (57.8)		195 (60.9)		81 (65.3)		56 (57.1)
Non-physician	23 787 (45.6)		700 (42.2)		125 (39.1)		43 (34.7)		42 (42.9)
Types of medical facility									
General hospital	11 130 (21.3)		159 (9.6)		22 (6.9)		6 (4.8)		10 (10.2)
Hospital and clinic	25 418 (48.7)		1104 (66.6)		226 (70.6)		91 (73.4)		69 (70.4)
Dental hospital and clinic	12 213 (23.4)		295 (17.8)		52 (16.3)		20 (16.1)		12 (12.2)
Others	3441 (6.6)		100 (6.0)		20 (6.3)		7 (5.7)		7 (7.1)
Area of medical facility									
Metropolitan	25 839 (49.5)		827 (49.9)		170 (53.1)		64 (51.6)		49 (50.0)
Urban	22 070 (42.3)		603 (36.4)		95 (29.7)		40 (32.3)		32 (32.7)
Rural	4293 (8.2)		228 (13.8)		55 (17.2)		20 (16.1)		17 (17.4)
Calendar year of birth									
<1960	8694 (16.7)		1088 (65.6)		228 (71.3)		89 (71.8)		74 (75.5)
1960–1969	18 469 (35.4)		380 (22.9)		71 (22.2)		29 (23.4)		20 (20.4)
≥1970	25 039 (48.0)		190 (11.5)		21 (6.6)		6 (4.8)		4 (4.1)
Calendar year of work begun									
<1996	9184 (17.6)		657 (39.6)		135 (42.2)		49 (39.5)		48 (49.0)
1996–2004	21 140 (40.5)		658 (39.7)		127 (39.7)		53 (42.7)		32 (32.7)
≥2005	21 878 (41.9)		343 (20.7)		58 (18.1)		22 (17.7)		18 (18.4)
Years of employment duration									
<1	7283 (14.0)		211 (12.7)		40 (12.5)		19 (15.3)		9 (9.2)
1–4	15 390 (29.5)		423 (25.5)		76 (23.8)		27 (21.8)		23 (23.5)
5–9	13 795 (26.4)		275 (16.6)		50 (15.6)		20 (16.1)		15 (15.3)
≥10	15 734 (30.1)		749 (45.2)		154 (48.1)		58 (46.8)		51 (52.0)
Cumulative badge dose (mSv)									
<1	21 651 (41.5)		568 (34.3)		102 (31.9)		42 (33.9)		31 (31.6)
1–4	12 103 (23.2)		315 (19.0)		70 (21.9)		30 (24.2)		17 (17.4)
5–19	10 246 (19.6)		284 (17.1)		45 (14.1)		16 (12.9)		13 (13.3)
20–49	5649 (10.8)		265 (16.0)		53 (16.6)		14 (11.3)		19 (19.4)
≥50	2553 (4.9)		226 (13.6)		50 (15.6)		22 (17.7)		18 (18.4)
Smoking status (pack-years)^a^									
Never smoker	19 552 (41.7)		435 (26.8)		75 (23.6)		23 (18.8)		23 (23.5)
Former smoker	12 589 (26.9)		811 (49.9)		176 (55.2)		71 (57.5)		56 (58.1)
Current smoker <12.5	7493 (16.0)		287 (17.7)		54 (16.9)		24 (19.2)		15 (15.3)
Current smoker ≥12.5	7198 (15.4)		91 (5.6)		14 (4.3)		6 (4.4)		3 (3.1)
Alcohol intake (per month)^a^									
Never	11 125 (23.8)		688 (42.4)		142 (44.5)		54 (43.3)		46 (47.0)
≤1	7979 (17.0)		224 (13.8)		43 (13.5)		16 (12.9)		14 (14.5)
2–3	16 765 (35.8)		387 (23.8)		71 (22.4)		29 (23.6)		19 (20.0)
≥4	10 963 (23.4)		325 (20.0)		62 (19.6)		25 (20.2)		18 (18.5)
Sleep duration (hour per day) ^a^									
<7	13 774 (29.4)		539 (33.2)		106 (33.3)		41 (33.0)		33 (34.0)
≥7	33 058 (70.6)		1085 (66.8)		212 (66.7)		83 (67.0)		64 (66.0)
Shift work ^a^									
Never	29 263 (62.5)		1029 (63.4)		200 (63.0)		77 (62.2)		61 (62.6)
Ever	17 569 (37.5)		595 (36.6)		118 (37.0)		47 (37.8)		36 (37.4)
Heart dose (mGy)		5.1 (0.003–301.8)		12.1 (0.003–217.8)		13.6 (0.003–204.1)		13.7 (0.003–204.1)		15.7 (0.003–136.9)
Thyroid dose (mGy)		9.8 (0.005–579.4)		23.2 (0.005–418.1)		26.0 (0.005–391.8)		26.2 (0.005–391.8)		30.1 (0.005–262.8)
Person years of follow-up		874 415		19 080		3499		1290		1162
Years of follow-up per person		16.8		11.5		10.9		10.4		11.9
Age at start of follow-up		34.2		49.6		52.2		51.6		54.5
Age at end of follow-up		50.9		61.1		63.1		62.0		66.4

a Variables obtained from multiple imputations by chained equations; numbers may not be sum to total due to unknown.

The results of the analysis of SMR of total deaths, CD, IHD, and CeVD are shown in table 2. The SMR of total deaths, CD, IHD, and CeVD were significantly lower among male workers than among the general population at 0.47 (95% CI 0.45–0.50), 0.54 (95% CI 0.48–0.60), 0.77 (95% CI 0.63–0.90), and 0.38 (95% CI 0.31–0.46), respectively. A decreased SMR was observed in all subgroups.

**Table 2:** Standardized mortality ratio (SMR) of mortality from all causes and circulatory disease among male diagnostic medical radiation workers in South Korea, 1996–2019. [CD=circulatory disease; CeVD=cerebrovascular disease; CI=confidence intervals; IHD=ischemic heart disease].

	Total deaths		CD		IHD		CeVD	
			
	Cases	SMR (95% CI)	Cases	SMR (95% CI)	Cases	SMR (95% CI)	Cases	SMR (95% CI)
Total	1658	0.47 (0.45–0.50)	320	0.54 (0.48–0.60)	124	0.77 (0.63–0.90)	98	0.38 (0.31–0.46)
Job title								
Physician	958	0.43 (0.40–0.46)	195	0.51 (0.43–0.58)	81	0.74 (0.58–0.91)	56	0.34 (0.25–0.43)
Non-physician	700	0.56 (0.52–0.60)	125	0.61 (0.50–0.71)	43	0.82 (0.57–1.06)	42	0.46 (0.32–0.60)
Types of medical facility								
General hospital	159	0.32 (0.27–0.37)	22	0.31 (0.18–0.44)	6	0.29 (0.06–0.52)	10	0.35 (0.13–0.57)
Hospital and clinic	1104	0.52 (0.49–0.55)	226	0.60 (0.52–0.68)	91	0.92 (0.73–1.11)	69	0.41 (0.31–0.51)
Dental hospital and clinic	295	0.43 (0.38–0.48)	52	0.47 (0.34–0.60)	20	0.61 (0.34–0.88)	12	0.27 (0.12–0.42)
Others	100	0.48 (0.38–0.58)	20	0.61 (0.34–0.88)	7	0.78 (0.20–1.35)	7	0.49 (0.13–0.85)
Area of medical facility								
Metropolitan	827	0.44 (0.41–0.47)	170	0.53 (0.45–0.61)	64	0.74 (0.56–0.93)	49	0.34 (0.24–0.43)
Urban	603	0.48 (0.44–0.52)	95	0.46 (0.37–0.56)	40	0.70 (0.48–0.92)	32	0.37 (0.24–0.50)
Rural	228	0.64 (0.55–0.73)	55	0.83 (0.60–1.06)	20	1.13 (0.61–1.65)	17	0.63 (0.33–0.94)
Calendar year of work begun								
<1996	657	0.47 (0.43–0.50)	135	0.52 (0.43–0.61)	49	0.73 (0.52–0.93)	48	0.41 (0.29–0.52)
1996–2004	658	0.49 (0.45–0.53)	127	0.57 (0.47–0.67)	53	0.85 (0.62–1.08)	32	0.34 (0.22–0.46)
≥2005	343	0.47 (0.42–0.52)	58	0.53 (0.39–0.66)	22	0.69 (0.40–0.97)	18	0.41 (0.22–0.60)
Years of employment duration								
<1	211	0.70 (0.60–0.80)	40	0.83 (0.58–1.09)	19	1.48 (0.81–2.15)	9	0.44 (0.15–0.72)
1–4	423	0.65 (0.58–0.71)	76	0.70 (0.54–0.86)	27	0.94 (0.59–1.30)	23	0.50 (0.28–0.71)
5–9	275	0.36 (0.31–0.40)	50	0.40 (0.29–0.52)	20	0.53 (0.29–0.78)	15	0.32 (0.16–0.48)
≥10	749	0.43 (0.40–0.46)	154	0.49 (0.41–0.57)	58	0.69 (0.51–0.87)	51	0.36 (0.26–0.46)
Cumulative badge dose (mSv)								
<1	568	0.52 (0.48–0.57)	102	0.57 (0.46–0.68)	42	0.84 (0.58–1.09)	31	0.42 (0.27–0.57)
1–4	315	0.42 (0.37–0.47)	70	0.59 (0.45–0.74)	30	0.87 (0.56–1.19)	17	0.34 (0.17–0.51)
5–19	284	0.44 (0.38–0.49)	45	0.42 (0.29–0.55)	16	0.52 (0.26–0.78)	13	0.28 (0.12–0.44)
20–49	265	0.50 (0.44–0.57)	53	0.55 (0.40–0.70)	14	0.58 (0.28–0.88)	19	0.44 (0.24–0.64)
≥50	226	0.47 (0.41–0.53)	50	0.53 (0.38–0.68)	22	0.97 (0.56–1.38)	18	0.40 (0.21–0.58)
Smoking status (pack-years)								
Never smoker	435	0.45 (0.41–0.50)	75	0.49 (0.38–0.61)	23	0.54 (0.32–0.76)	23	0.36 (0.21–0.52)
Former smoker	811	0.51 (0.48–0.55)	176	0.59 (0.50–0.68)	71	0.93 (0.71–1.14)	56	0.42 (0.31–0.53)
Current smoker <12.5	287	0.48 (0.42–0.53)	54	0.54 (0.39–0.68)	24	0.83 (0.49–1.16)	15	0.35 (0.17–0.53)
Current smoker ≥12.5	91	0.43 (0.33–0.54)	14	0.49 (0.21–0.78)	6	0.66 (0.08–1.23)	3	0.31 (0.01–0.66)

The RR of CD mortality was significantly higher among non-physicians, workers at hospitals and clinics, and those working in rural areas. The CD mortality risks were significantly increased for the groups with cumulative radiation doses of 20–49 and ≥50 mSv. Analysis results for CD, IHD, and CeVD mortality associated with additional adjustments for non-radiation risk factors (smoking status, alcohol intake, sleep duration, and shift work) did not reveal any significant differences from the main results (table 3).

**Table 3:** Relative risk of circulatory disease (CD) mortality among male diagnostic medical radiation workers in South Korea, 1996–2019. [CeVD=cerebrovascular disease; CI=confidence intervals; IHD=ischemic heart disease; RR=relative risk].

	CD		IHD		CeVD	
		
	RR ^a^(95% CI)	RR ^b^(95% CI)	RR ^a^(95% CI)	RR ^b^(95% CI)	RR ^a^(95% CI)	RR ^b^(95% CI)
Job title						
Physician	1.00 (Reference)	1.00 (Reference)	1.00 (Reference)	1.00 (Reference)	1.00 (Reference)	1.00 (Reference)
Non-physician	1.27 (1.00–1.62)	1.38 (1.08–1.76)	1.12 (0.75–1.66)	1.22 (0.82–1.82)	1.52 (0.98–2.34)	1.61 (1.04–2.49)
Types of medical facility						
General hospital	1.00 (Reference)	1.00 (Reference)	1.00 (Reference)	1.00 (Reference)	1.00 (Reference)	1.00 (Reference)
Hospital and clinic	2.14 (1.37–3.35)	1.88 (1.20–2.94)	3.33 (1.44–7.70)	2.85 (1.24–6.57)	1.24 (0.62–2.47)	1.12 (0.56–2.22)
Dental hospital and clinic	1.63 (0.98–2.72)	1.42 (0.84–2.39)	2.23 (0.88–5.67)	1.92 (0.75–4.93)	0.78 (0.33–1.85)	0.69 (0.28–1.66)
Others	1.85 (1.00–3.41)	1.67 (0.90–3.09)	2.45 (0.81–7.37)	2.06 (0.68–6.19)	1.31 (0.49–3.50)	1.20 (0.45–3.20)
Area of medical facility						
Metropolitan	1.00 (Reference)	1.00 (Reference)	1.00 (Reference)	1.00 (Reference)	1.00 (Reference)	1.00 (Reference)
Urban	0.85 (0.66–1.10)	0.86 (0.66–1.10)	0.94 (0.63–1.40)	0.95 (0.64–1.41)	1.04 (0.66–1.63)	1.05 (0.67–1.64)
Rural	1.64 (1.21–2.22)	1.64 (1.21–2.23)	1.62 (0.98–2.69)	1.63 (0.98–2.70)	1.75 (1.00–3.04)	1.76 (1.01–3.06)
Calendar year of work begun						
<1996	1.00 (Reference)	1.00 (Reference)	1.00 (Reference)	1.00 (Reference)	1.00 (Reference)	1.00 (Reference)
1996–2004	0.66 (0.44–0.97)	0.64 (0.44–0.95)	0.81 (0.43–1.49)	0.78 (0.42–1.45)	0.43 (0.20–0.93)	0.43 (0.20–0.92)
≥2005	0.40 (0.24–0.66)	0.40 (0.24–0.66)	0.45 (0.20–1.00)	0.44 (0.20–0.99)	0.32 (0.12–0.85)	0.33 (0.13–0.86)
Years of employment duration						
<1	1.00 (Reference)	1.00 (Reference)	1.00 (Reference)	1.00 (Reference)	1.00 (Reference)	1.00 (Reference)
1–4	0.93 (0.63–1.36)	0.93 (0.63–1.30)	0.66 (0.37–1.19)	0.66 (0.37–1.19)	1.32 (0.61–2.85)	1.32 (0.61–2.86)
5–9	0.57 (0.37–0.86)	0.57 (0.37–0.87)	0.43 (0.23–0.82)	0.43 (0.23–0.82)	0.85 (0.37–1.98)	0.86 (0.37–1.98)
≥10	0.66 (0.46–0.94)	0.69 (0.49–0.99)	0.47 (0.28–0.81)	0.50 (0.29–0.84)	1.04 (0.51–2.14)	1.08 (0.53–2.22)
Cumulative badge dose (mSv)						
<1	1.00 (Reference)	1.00 (Reference)	1.00 (Reference)	1.00 (Reference)	1.00 (Reference)	1.00 (Reference)
1–4	1.54 (1.09–2.17)	1.52 (1.08–2.17)	1.64 (0.95–2.84)	1.63 (0.94–2.83)	1.13 (0.58–2.19)	1.12 (0.58–2.17)
5–19	1.20 (0.79–1.83)	1.23 (0.81–1.87)	1.04 (0.52–2.09)	1.06 (0.53–2.12)	1.05 (0.48–2.29)	1.07 (0.49–2.33)
20–49	1.79 (1.12–2.85)	1.93 (1.21–3.09)	1.22 (0.55–2.74)	1.33 (0.59–3.00)	1.71 (0.73–4.00)	1.82 (0.78–4.25)
≥50	1.78 (1.09–2.90)	1.95 (1.20–3.19)	2.00 (0.93–4.29)	2.20 (1.02–4.76)	1.66 (0.69–3.99)	1.76 (0.73–4.28)

a Adjusted for attained age (<40, 5-year intervals from age 40–84, ≥85 years), birth year (<1960, 1960–1969, ≥1970), and years of employment duration (<1, 1–4, 5–9, ≥10).

b Adjusted for attained age (<40, 5-year intervals from age 40–84, ≥85 years), birth year (<1960, 1960–1969, ≥1970), and years of employment duration (<1, 1–4, 5–9, ≥10), smoking status (never, former, current <12.5, current ≥12.5 pack-years, unknown), alcohol intake (never, ≤1, 2–3, ≥4 per month, unknown), sleeping (<7, ≥7 hour per day, unknown), and shift work (never, ever, unknown).

Linear dose–response estimates (ERR/100 mGy) for mortality from CD, IHD, and CeVD associated with the cumulative heart dose or thyroid dose with different lags are summarized in table 4. A linear dose–response model provided an estimated ERR per 100 mGy for CD (ERR/100 mGy=0.85, 95% CI -0.11–1.82), IHD (ERR/100 mGy=1.18, 95% CI -0.69–3.05), and CeVD (ERR/100 mGy=0.23, 95% CI -0.48–0.94). After adjusting for the imputed non-radiation risk factors, the magnitude of the measured association remained similar to the main findings.

**Table 4:** Excess relative risk per 100 mGy of circulatory disease mortality by cumulative organ doses and lag years among male diagnostic medical radiation workers in South Korea, 1996–2019. [CD=circulatory disease; CeVD=cerebrovascular disease; CI=confidence intervals; ERR=excess relative risk; IHD=ischemic heart disease].

	All workers (N=53 860)	

	ERR/100 mGy ^a^(95% CI)	ERR/100 mGy ^b^(95% CI)
10-years lag	
CD ^c^	0.85 (-0.11–1.82)	0.81 (-0.11–1.74)
IHD ^c^	1.18 (-0.69–3.05)	1.16 (-0.67–2.99)
CeVD ^d^	0.23 (-0.48–0.94)	0.27 (-0.41–0.96)
15-years lag	
CD ^c^	0.84 (-0.13–1.81)	0.78 (-0.14–1.70)
IHD ^c^	1.18 (-0.72–3.07)	1.17 (-0.69–3.03)
CeVD ^d^	0.15 (-0.50–0.80)	0.19 (-0.43–0.8)
5-years lag	
CD ^c^	0.83 (-0.11–1.78)	0.86 (-0.08–1.80)
IHD ^c^	1.11 (-0.69–2.91)	1.13 (-0.65–2.92)
CeVD ^d^	0.27 (-0.47–1.00)	0.37 (-0.39–1.12)
Without lag	
CD ^c^	0.85 (-0.09–1.80)	0.90 (-0.05–1.85)
IHD ^c^	1.29 (-0.62–3.20)	1.31 (-0.59–3.22)
CeVD ^d^	0.26 (-0.46–0.99)	0.36 (-0.39–1.11)

a Adjusted for attained age (<40, 5-year intervals from age 40–84, ≥85 years), birth year (<1960, 1960–1969, ≥1970), and years of employment duration (<1, 1–4, 5–9, ≥10).

b Adjusted for attained age (<40, 5-year intervals from age 40–84, ≥85 years), birth year (<1960, 1960–1969, ≥1970), and years of employment duration (< 1, 1–4, 5–9, ≥10), smoking status (never, former, current <12.5, current ≥12.5 pack-years, unknown), alcohol intake (never, ≤1, 2–3, ≥4 per month, unknown), sleeping (<7, ≥7 hour per day, unknown), and shift work (never, ever, unknown).

c Analysis based on heart dose.

d Analysis based on thyroid dose.

Sensitivity analyses restricted to workers who started work after 1995, were employed for ≥1 year, or had a cumulative badge dose of ≥1 mSv showed no meaningful difference compared with the overall results (table 5). Additionally, the risk estimates from exposure to occupational radiation doses did not show significant differences by job title (physician versus non-physician).

**Table 5:** Sensitivity analyses of excess relative risks (ERR) per 100 mGy of circulatory disease mortality by study populations and lag years among male diagnostic medical radiation workers in South Korea, 1996–2019. [CD=circulatory disease; CeVD=cerebrovascular disease; CI=confidence intervals; IHD=ischemic heart disease].

	Workers started job ≥1996 (N=44 019)	Workers employed ≥1 years (N=46 366)	Workers had ≥1 mSv (N=31 641)	Job title	

				Physician (N=29 373)	Non-physician (N=24 487)
				
	ERR/100 mGy a(95% CI)	ERR/100 mGy a(95% CI)	ERR/100 mGy a(95% CI)	ERR/100 mGy a(95% CI)	ERR/100 mGy a(95% CI)
10-years lag					
CD b	2.33 (-5.27–9.94)	0.36 (-0.27–1.00)	0.54 (-0.29–1.37)	0.80 (-0.76–2.36)	0.87 (-0.14–1.88)
IHD b	1.08 (-9.79–11.95)	0.60 (-0.63–1.84)	0.99 (-0.82–2.80)	1.17 (-1.87–4.20)	1.19 (-0.75–3.13)
CeVD c	-0.11 (-5.58–5.35)	0.11 (-0.42–0.65)	0.18 (-0.51–0.88)	-0.14 (-0.92–0.63)	0.30 (-0.46–1.06)
15-years lag					
CD b	2.29 (-9.22–13.79)	0.36 (-0.29–1.02)	0.53 (-0.31–1.38)	0.92 (-0.73–2.57)	0.82 (-0.19–1.82)
IHD b	-0.38 (-5.48–4.72)	0.61 (-0.67–1.89)	1.03 (-0.85–2.91)	1.30 (-1.93–4.52)	1.13 (-0.81–3.07)
CeVD c	-0.15 (-8.48–8.17)	0.07 (-0.45–0.59)	0.11 (-0.54–0.75)	-0.16 (-0.92–0.61)	0.21 (-0.50–0.92)
5-years lag					
CD b	2.53 (-3.62–8.67)	0.35 (-0.27–0.98)	0.53 (-0.29–1.35)	0.75 (-0.75–2.25)	0.86 (-0.13–1.85)
IHD b	1.43 (-7.48–10.34)	0.56 (-0.63–1.75)	0.90 (-0.80–2.60)	1.01 (-1.82–3.83)	1.16 (-0.72–3.05)
CeVD c	-0.04 (-4.57–4.49)	0.14 (-0.40–0.68)	0.23 (-0.50–0.96)	-0.05 (-0.92–0.83)	0.31 (-0.46–1.08)
Without lag					
CD b	3.27 (-2.64–9.19)	0.36 (-0.26–0.98)	0.55 (-0.27–1.38)	0.71 (-0.75–2.17)	0.90 (-0.10–1.90)
IHD b	3.60 (-6.30–13.50)	0.63 (-0.58–1.84)	1.03 (-0.76–2.82)	1.14 (-1.77–4.05)	1.35 (-0.66–3.35)
CeVD c	-0.16 (-3.70–3.39)	0.14 (-0.40–0.68)	0.24 (-0.50–0.97)	-0.04 (-0.91–0.84)	0.32 (-0.45–1.09)

a Adjusted for attained age (<40, 5-year intervals from age 40–84, ≥85 years), birth year (<1960, 1960–1969, ≥1970), and years of employment duration (<1, 1–4, 5–9, ≥10).

b Analysis based on heart dose.

c Analysis based on thyroid dose.

## Discussion

Our findings revealed that occupational radiation doses were not significantly associated with CD mortality among male diagnostic medical radiation workers in South Korea between 1996 and 2019. The non-statistically significant positive ERR findings for CD, IHD, and CeVD were similar between the study populations, alternative lag-years, and adjusted for lifestyle factors. Considering the growing number of medical radiation workers and that currently available scientific inferences on the dose–response for CD are controversial and inconsistent, it is essential to investigate the risk of CD among medical radiation workers exposed to chronic low-dose radiation by conducting a study with extended follow-up, together with consideration of other risk factors.

Our non-significant findings of dose–response relationships between occupational radiation doses and CD mortality may be related to cohort characteristics such as young workers with a short job duration. Our cohort’s population was younger in age than medical radiation workers in other studies, which reported statistically significant CD risk. Considering that the frequency of CD increases with age, our cohort may not have reached the age when these outcomes occur. A large proportion of young workers in our cohort started working after the 1990s. This leads to a short follow-up and yields a lower cumulative radiation dose in comparison to other cohorts, which likely resulted in a limited statistical power to detect a weak association with cumulative low-dose radiation exposure. The estimated statistical power for CD deaths in this study was low (7.1%) based on a one-side test of trend using the previous methods (23, 24) (supplementary table S2), and the findings needed to be interpreted cautiously. Despite the limited power, there is value in studies of low-dose populations and there is a need for efforts to consider the consistency of results from low-dose study and the results of moderate- and high-dose studies (24). Continued follow-up the workers with updated registry doses and to expand the cohort by including workers who enrolled in the NDR system after 2011 will improve the precision of the risk estimates.

Our non-significant positive dose–response estimates of CD with occupational radiation doses were generally comparable to those seen amongst medical radiation workers in the United States (12); in the Korean study based on morbidity among 11 500 diagnostic medical radiation workers (11); and amongst Mayak nuclear workers in Russia (20). However, there is considerable variation in the risk estimates from specific studies. Significant dose–response associations have been observed for cardiovascular disease in Canadian radiation workers (25), and in the International Nuclear Workers Study (INWORKS) of three large national groups of workers in France, the United Kingdom (UK), the United States (US) (26), as well as in the National Registry for Radiation Workers (NRRW) in the UK (27, 28), whereas non-significant negative estimates for CD were observed in German uranium miners (29), US astronauts (30), and French uranium workers involved in nuclear fuel production cycle (31). The differences in the findings regarding the risk of CD after occupational radiation exposures across studies could be explained by several factors, such as differences in baseline risks, heterogeneity between incidence and mortality, possible internal radiation exposure, non-radiation confounding factors, and differences between exposure circumstances among the cohorts (32). Additionally, statistical fluctuations may also contribute to the different findings of the studies.

Our study showed a slightly stronger dose–response relationship with IHD in comparison to CeVD, this finding differed with results from previous studies conducted on other radiation workers. Meta-analysis has reported that CeVD has a larger risk (ERR/Sv=0.236, 95% CI 0.062–0.410) than IHD (ERR/Sv=0.082, 95% CI 0.057– 0.106) (6). Among recently reported findings of IHD and CeVD risk estimates derived among medical radiation workers in the US, there was no significant difference in the IHD and CeVD (ERR/100 mGy= -0.10, 95% CI -0.27–0.06; ERR/100 mGy=0.04, 95% CI -0.16–0.23, respectively) (12). On the other hand, in a cohort of atomic bomb survivors (18), stronger and significant dose–response associations have been reported for mortality of heart diseases or hypertension compared with those for stroke. The pattern of ERR estimates by CD subtypes in this mortality study was also somewhat different from the results of our previous subset population based on morbidity, which showed a higher risk of CeVD than IHD (11). However, cautious interpretation is needed because the previous morbidity study included both male and female workers who completed a questionnaire survey and were linked with National Health Insurance Service data (N=11 500), whereas this study included male workers enrolled in the NDR data between 1996 and 2011 (N=53 860). Our previous morbidity study also showed a higher risk of CeVD than this mortality study. The longer latency for mortality compared with incidence may be one of the possible reasons. The difference in the specific pathogenesis of radiation-induced CeVD (ie, incidence leading to chronic rather than acute forms of CeVD) could also be a potential reason (33). Some diversity in dose–response effects by heart disease subtype and by outcome index needs to be further studied with a longer follow-up in this cohort.

The significantly lower CD mortality observed in our diagnostic medical radiation workers in comparison to the general population reflects the classical, healthy worker effect. The findings showed comparable healthy worker effects to those of other medical radiation worker studies in the US (12) and Canada (25). This may be due to the large proportion of young workers in our study cohort. The findings of significantly higher RR for CD mortality among those who worked in hospitals or clinics and rural areas may imply that the presence of a risk factor other than radiation may be responsible for increasing CD mortality in these subgroups; this warrants the need for further studies that investigate more risk factors among medical radiation workers.

This study had some limitations. Firstly, our study used mortality data, which have more limited information than incidence, to identify the association with radiation exposure and CD. Secondly, direct information on non-radiation lifestyle-related CD risk factors was unavailable, as was the case in most other occupational studies. Although we supplemented non-radiation risk factor information through multiple imputations, it was limited to a few risk factors and we could not include other important factors such as body mass index, dietary factors, and disease history. Thirdly, our study included relatively young cohort members, a short follow-up period, and narrow and lower dose distributions which resulted in low statistical power (24). Finally, there is uncertainty in estimating organ doses because we applied an organ-specific conversion coefficient in general radiology exposure scenarios for all workers. Even so, 8% of medical workers have been involved in fluoroscopically-guided interventional procedures in South Korea (34) and the specific coefficients determined by exposure scenario should be applied for more exact estimation in the future.

In summary, we examined CD mortality in a cohort of Korean diagnostic medical radiation workers exposed to ionizing radiation. Our results showed a statistically non-significant positive association between occupational radiation doses and CD mortality. Nevertheless, with the increasing use of radiation in modern medical practices and high baseline rates of CD, it is essential to investigate continuously the risk of CD in workers exposed to chronic low-dose radiation.

### Funding

The National Research Foundation of Korea (NRF) supported this work through a grant funded by the Korean government (MSIT) (No. 2020R1A2C1008891).

### Conflict of interest

All authors declare that they have no conflict of interest. The funders had no role in the design of the study; the collection, analyses, or interpretation of data; the writing of the manuscript; or the decision to publish the results.

## Supplementary material

Supplementary material
