# Supplementary material for: Circulatory disease mortality among male medical radiation workers in South Korea, 1996–2019
Source: Scand J Work Environ Health. 2023 Feb 27;49(2):99–107. doi: 10.5271/sjweh.4066 (PMC10577014; doi:10.5271/sjweh.4066)
Supplement: Supplementary material [file SJWEH-49-99-S001.docx]

**Circulatory disease mortality among male medical radiation workers in South Korea, 1996–2019^1^**

*by Ye Jin Bang, MPH,^1, 2^ Young Min Kim, PhD,^3^ Won Jin Lee, MD, PhD ^1, 2^*

Correspondence to: Won Jin Lee, MD, MPH, PhD, Department of Preventive Medicine, Korea University College of Medicine, 73, Goryeodae-ro, Seongbuk-gu, Seoul 02841, South Korea. [E-mail: leewj@korea.ac.kr]

**Supplemental table S1. Estimated dose coefficients in linear and linear-quadratic excess relative risk models (lagged by 10-years) among male diagnostic medical radiation workers in South Korea, 1996-2019**

|  | Linear model |  | Linear-quadratic model | |  | P curb^a^ |
| --- | --- | --- | --- | --- | --- | --- |
|  | Linear dose coefficient  (95% CI) |  | Linear dose coefficient  (95% CI) | Quadratic dose coefficient  (95% CI) |  |  |
| CD^b^ | 0.85 (-0.11–1.82) |  | 1.65 (-0.37–3.68) | -0.67 (-1.94–0.60) |  | 0.128 |
| IHD^b^ | 1.18 (-0.69–3.05) |  | 2.03 (-1.54–5.59) | -0.82 (-3.05–1.41) |  | 0.520 |
| CeVD^c^ | 0.23 (-0.48–0.94) |  | 0.37 (-1.18–1.92) | -0.07 (-0.72–0.59) |  | 0.460 |

^a^Likelihood ratio test for quadratic departure from linearity; ^b^analysis based on heart dose; ^c^analysis based on thyroid dose.

CD, circulatory disease; CeVD, cerebrovascular disease; CI, confidence intervals; IHD, ischemic heart disease.

**Supplemental table S2. Mean cumulative badge doses and person years by dose range for power calculations of circulatory disease mortality among male diagnostic medical radiation workers in South Korea, 1996–2019**

| Dose range (mSv) | Mean dose (mSv) | Person years |
| --- | --- | --- |
| Total | 10.6 | 893 496 |
| < 1 | 0.3 | 297 602 |
| 1–4 | 2.4 | 212 088 |
| 5–19 | 10.6 | 201 046 |
| 20–49 | 31.6 | 122 083 |
| ≥ 50 | 85.0 | 60 678 |
| Power (%)^a^ | 7.1 | |

^a^We estimated the power of a one-sided test of trend with type I error of 5% using the methodology described by Little et al (2010) and Gilbert et al (2020), using 100 000 Monte Carlo samples. The model is fitted via Poisson maximum likelihood.
